# Statistical analysis plan: Early mobilization by head-up tilt with stepping versus standard care after severe traumatic brain injury

**DOI:** 10.1016/j.conctc.2021.100856

**Published:** 2021-11-15

**Authors:** Christian Gunge Riberholt, Christian Gluud, Janus Christian Jakobsen, Christian Ovesen, Jesper Mehlsen, Kirsten Møller

**Affiliations:** a Department of Neurorehabilitation / Traumatic Brain Injury Unit, Rigshospitalet, University of Copenhagen, Kettegard Alle 30, 2650, Hvidovre, Denmark; b Department of Clinical Medicine, Faculty of Health and Medical Sciences, University of Copenhagen, Denmark; c Copenhagen Trial Unit, Centre for Clinical Intervention Research, Dept 7812, Rigshospitalet, Copenhagen University Hospital, Blegdamsvej 9, DK-2100 Copenhagen, Denmark; d Department of Regional Health Research, The Faculty of Heath Sciences, University of Southern Denmark, Campusvej 55, 5230, Odense, Denmark; e Department of Cardiology, Holbæk Hospital, Smedelundsgade 60, DK-4300, Holbæk, Denmark; f Department of Neurology, Bispebjerg and Frederiksberg Hospital, Bispebjerg Bakke 23, 2400, Copenhagen, Denmark; g Surgical Pathophysiology Unit, Juliane Marie Centre, Rigshospitalet, Copenhagen University Hospital, Tagensvej 75, DK-2100, Copenhagen Ø, Denmark; h Department of Neuroanaesthesiology, Rigshospitalet, University of Copenhagen, Blegdamsvej 9, 2100, København Ø, Denmark

**Keywords:** Statistical analysis plan, Early mobilization, Trial sequential analysis, Traumatic brain injury, AE, adverse event, AR, adverse reaction, CG, Control group, 95% CI, 95% confidence interval, CRS-R, Coma Recovery Scale-Revised, EFA, Early Functional Ability, EOE, Early orthostatic exercise, FIM, Functional Independence Measure, GCS, Glasgow coma scale, ICU, Intensive care unit, SAE, serious adverse event, SAR, serious adverse reaction, SD, standard deviation, SUSAR, suspected unexpected adverse reaction

## Abstract

**Background:**

Early mobilization on a tilt table with stepping versus standard care may be beneficial for patients with severe brain injury, but data from randomized clinical trials are lacking. This detailed statistical analysis plan describes the analyses of data collected in a randomized clinical feasibility trial for early mobilization by head-up tilt with stepping versus standard care after severe traumatic brain injury.

**Methods:**

Primary feasibility outcomes are the proportion of included participants who were randomized out of all screened patients; the proportion of participants allocated to the experimental intervention who received at least 60% of the planned exercise sessions; and safety outcomes such as adverse events and reactions and serious adverse events and reactions. Exploratory clinical outcomes are suspected unexpected serious adverse reactions; and functional outcomes as assessed by the Coma Recovery Scale-Revised at four weeks; Early Functional Ability Scale and Functional Independence Measure at three months. The description includes the statistical analysis plan, including the use of multiple imputations and Trial Sequential Analysis.

**Table:** SAP revision history

SAP version	Action	Changes made	Timing of SAP	Date changed
1.0	Submitted for Trials	Original first submission	Before the last three-month follow-up and before starting analysis	March 12, 2019
1.1	Editorial revision	Added information to the manuscript and submitted checklist	Last three-month follow-up and analysis completed	11th of June 2019
1.2	First revision	Changes made in response to reviewer comments	All analysis finished according to protocol except per-protocol analysis.	December 23, 2019
1.3	Rejected	The reviewer pointed out unclarity in the definition of the feasibility outcomes and lacked specification in the analysis of the data.	All analysis finished according to the statistical analysis plan	April 19, 2020
1.4	Submitted for Brain Injury	Changes made in response to the unclarity pointed out by the reviewer	All analysis finished according to the statistical analysis plan	June 1, 2020
1.5	Submitted for Contemporary Clinical Trials	Adjusted according to journal criteria	All analysis finished according to the statistical analysis plan	November 19, 2020

## 1 Introduction

The early mobilization by head-up tilt with stepping versus standard care after severe traumatic brain injury (HUT-TBI) trial is a randomized clinical trial assessing the feasibility of using a tilt-table with integrated stepping for early mobilization to the upright position in the neuro-intensive care unit [1]. The possible negative effects of bed rest on human physiology have been investigated for decades [[2], [3], [4]]. With the possibility of counteracting the adverse effects of prolonged bed rest, it might be beneficial for the patients to undergo early mobilization, whereby they are moved to the upright position using a tilt table. The simultaneous stepping is intended to counteract orthostatic hypotension in the standing position.

Early rehabilitation of patients with a severe traumatic brain injury has hitherto been subject to few studies, in which the interventions have been incompletely described [5,6]. Nonetheless, the available studies indicate that early mobilization may improve functional outcome after traumatic brain injury. However, a large randomized clinical trial, the AVERT trial, showed no benefit of early and intensive mobilization on functional outcomes measured three months after stroke [7]. Moreover, a systematic review with a meta-analysis found no impact of early active mobilization and rehabilitation on mortality at discharge from the intensive care unit (ICU) in a large variety of non-neurological ICU patients. However, the intervention did increase muscle strength, walking ability, and the number of days alive and out of the hospital at six months [8].

The present trial assessed if using a tilt table for early orthostatic exercise was feasible in a group of patients with severe traumatic brain injury [1]. Here we report the detailed statistical analysis plan for the HUT-TBI trial [1], which has been updated and finalized during the data collection period. Besides the primary outcomes related to feasibility, the analysis plan also addresses the statistical handling of exploratory clinical outcomes.

## 2 Methods

### 2.1 Ethical approval

This randomized clinical feasibility trial was approved by the Scientific-Ethics Committee of the Capital Region (H-16041794) and is registered on www.clinicaltrials.gov (ClinicalTrials.gov identifier: NCT02924649); the trial protocol has been published in Trials [1]. The project manager (CGR) is responsible for collecting and storing data and all correspondence. After a patient was found to be eligible for the trial, informed consent from the proxy and a trial guardian (a physician not involved in the trial) was obtained by CGR. The trial was carried out following the principles of the Helsinki Declaration [9].

### 2.2 Primary research questions

Is an early head-up tilt protocol feasible in patients with severe traumatic brain injury, in terms of the number of participants who are successfully included, the number of exercise sessions performed in the experimental group, and the number of patients with serious adverse events (SAE) and non-serious adverse events (AE) and serious adverse reactions (SAR) and non-serious adverse reactions (AR)?

### 2.3 Exploratory research questions

• Does early head-up tilt with stepping reduce the number of AE, AR, SAE, and SAR compared with standard care after severe traumatic brain injury?
• Does early head-up tilt with stepping improve the level of consciousness (Coma Recovery Scale-Revised) after four weeks, early functional abilities (Early Functional Ability scale) after three months, or functional independence (Functional Independence Measure) after three months, compared with standard care after severe traumatic brain injury?
• Does head-up tilt with stepping improve the level of consciousness (Coma Recovery Scale-Revised), early functional abilities (Early Functional Ability scale), or functional independence (Functional Independence Measure) after one year compared with standard care in patients with severe traumatic brain injury?

## 3 Main trial design

The present statistical analysis plan describes our planned analyses for the feasibility trial, investigating head-up tilt with stepping versus standard care in patients with severe traumatic brain injury. As described in the published protocol, the sample size (n = 60) was chosen as a realistic number to reach for this feasibility trial [1]. No formal power calculation for efficacy was conducted as there was no information on which to base this calculation, and because we only wanted to examine the feasibility.

The trial is a randomized clinical feasibility trial with a pragmatic stratification according to the Glasgow Coma Score at inclusion (3–6 compared to 7–10 points). The patients are randomized in a 1:1 ratio by the Copenhagen Trial Unit using a central web-based randomization system.

Besides standard care, the experimental intervention group received daily (Monday to Friday) mobilization on a tilt-table to the standing position for up to 20 min per session. This orthostatic exercise continued for four weeks from randomization or until the patient could stand from a chair or bed with assistance. The tilt-table has a build-in stepping device that increases the venous return of blood to the heart and thereby counteracts orthostatic hypotension and increases standing time [10,11]. The control group received standard care. Standard care was decided in collaboration between doctors, nurses, and physiotherapists and was monitored during the trial. The standard care group used little time on mobilizing the patient to the edge of the bed or chair while admitted to the neurologic ICU. The focus of the physiotherapist is on respiratory function and in bed positioning to avoid bedsores.

## 4 Primary feasibility outcomes

Our primary feasibility outcomes are as follows:

The lower limit of the confidence interval of the inclusion ratio (the proportion of included participants randomized compared to all eligible patients). For example, if 44 of 60 eligible patients agree to participate, then the proportion will be 73% with a 95% confidence interval (95% CI) between 60% and 84%. The lower limit for this feasibility outcome is set at 60%; if the lower limit of the confidence interval of the gathered data of the HUT-TBI Trial is at 60% or higher, then the trial is successful in terms of inclusion. This is equivalent to a one-sided test (please see the statistical section below).

The lower limit of the confidence interval of the intervention success rate defined as the proportion of participants allocated to the experimental intervention who received at least 60% of the planned exercise sessions. For example, if 21 of 30 participants (70%) randomized to the experimental intervention group receive 60% of the exercise sessions, the lower limit of the confidence interval will be 52%. Accordingly, if the lower limit of the confidence interval of the gathered data of the HUT-TBI Trial is at or above 52%, the trial will be successful in terms of exercise completeness.

Both the inclusion ratio and the intervention success rate limits are arbitrary limits decided together with the clinical staff at the department. It, therefore, emphasizes clinical reality on the validity of the data.

Our safety outcomes are defined as either proportion of participants with either an SAE, SAR, AE, or AR not considered serious [12]. SAEs are defined as any undesirable event that results in death, is life-threatening, requires prolongation of existing hospitalization, results in persistent or significant disability or incapacity, or requires intervention to prevent permanent impairment or damage, whether considered related to the trial intervention or not [12]. AEs are defined as any undesirable event not considered serious occurring to a participant during the trial. The proportion of participants with at least one SAE, SAR, AR, or AE during the intervention period will be compared between the two intervention groups (please see the statistical section below).

## 5 Exploratory clinical outcomes

For the exploratory clinical outcomes, we have chosen three outcomes: The Coma Recovery Scale-Revised (CRS-R) [13], the Early Functional Ability scale (EFA) [14,15], and the Functional Independence Measure (FIM) [16,17], all of which are scored at baseline and after four-weeks, three-months and one-year. The CRS-R reflects changes in consciousness and will be analyzed at the four-week time point (end of the intervention period) comparing the two intervention groups and was scored by assessors blinded to the intervention allocation. The EFA evaluates early functional changes, and the FIM evaluates the ability to perform functions and activities of daily living independently. Both were evaluated at the three-month time point. Secondly, the data for all three exploratory clinical outcomes will be presented as longitudinal data in a figure (error bar plot) showing the mean and the 95% CI for each group. At the one-year follow-up, the same three outcome scales are used and supplemented by the Glasgow Outcome Scale – Extended (GOSE); the latter is used routinely at the department for the one-year follow-up.

### 5.1 Statistical analyses

#### 5.1.1 Statistical analysis will be handled using STATA (StataCorp, College Station, Texas, USA)

All baseline characteristics will be presented for each intervention group. Continuous variables will be summarized using means and standard deviations or medians and interquartile range depending on the distribution of data. Discrete variables will be presented as frequencies, proportions, and percentages.

The timing of outcome assessments can be found in the published protocol [1].

Regarding the feasibility outcome, we will not adjust for multiplicity since all three outcomes should be achieved for the trial to be considered feasible. That is, the inclusion ratio and exercise success rate should be above the decided limits, and the adverse events should not be significantly different in favor of the standard care group. The inclusion ratio and the exercise success ratio will be calculated as proportions including 95% CI using Wilsons interval and Jeffrey's interval [18]. If there is a difference between these two the most conservative estimate will be used. We have decided to use a one-sided test for the feasibility outcomes corresponding to the description above. In these analyses, a significance level of 2.5% will be used.

All our analyses will primarily be intention-to-treat, i.e., all randomized participants will be included in the primary analyses and analyzed as randomized. We will secondly perform per-protocol analyses, including the participants allocated to the intervention who received at least 60% of the planned exercise sessions compared to the patients in the standard care group.

If we do not reach the desired number of participants in the trial, we will consider analyzing our data using Trial Sequential Analysis [19,20]. In this case, we will use the pre-specified standard deviations and minimal relevant differences described in Supplementary Table 1 for the continuous outcome and the proportion in the control group for dichotomized outcomes. The calculations will be based on an alpha of 5%, a beta of 10% and for dichotomous outcome a relative risk reduction of 20%, while continuous outcomes will use the calculated variance from the trials control group. Trial Sequential Analysis reduces the risk of type I and type II errors due to small sample size and multiple outcome testing [20].

The analysis will start after the last three-month follow-up has been collected and after submission of this statistical analysis plan (end of March 2019). The analysis of the one-year follow-up data will start after data from the last patient has been collected in late December 2019.

## 6 Feasibility outcomes

The first two primary feasibility outcomes will be derived from the trial with the above-mentioned lower limits of the proportions. For the intervention to be feasible, both feasibility outcomes should be achieved, and the early orthostatic intervention group should not have an overrepresentation of SAE, AE, SAR, AR, or suspected unexpected serious adverse reactions (SUSAR).

All analyses described below using general linear regression, logistic regression, or mixed-model linear regression will be adjusted for the protocol specified stratification variable (high or low GCS).

We will use the inspection of data (descriptive analysis) to evaluate adverse events due to the low power. Secondly, the proportions of participants with one or more SAEs, SARs, ARs, and AEs between the two groups will be examined using Fisher's exact test [1]. Accordingly, we will use an alpha of 5%. Each patient with at least one SUSAR during the intervention period will be analyzed as an exploratory feasibility outcome, also using logistic regression analysis. Where appropriate, we will present data with a 95% CI.

## 7 Exploratory clinical outcomes

All exploratory clinical outcomes and physiological outcomes are on a continuous interval scale.

The exploratory clinical outcomes will primarily be compared between allocation groups at specified time points. The CRS-R will be analyzed at the four-week time point, and EFA and FIM will be analyzed at the three-month time point using general linear regression analysis.

Each outcome, with the corresponding minimal relevant difference, standard deviation, and power level, can be found in Additional File 1. The one-year follow up data for CRS-R, EFA, and FIM will be analyzed in the same way. Furthermore, for the one-year analysis, the Glasgow outcome scale extended will be compared between groups using general linear regression and adjusting for stratification-specific variables.

In case the regression models described above (linear regression and mixed model) cannot be fitted due to breach of their underlying assumptions (e.g., skewed distribution of data/residuals), non-parametric methods (e.g., Van Elteren's test) taking the stratified randomization into account will be employed. The analysis will, in all cases, be conducted at the pre-specified time points as stipulated above. As described in our protocol, we have still reported that all results will be interpreted as hypothesis-generating.

### 7.1 Missing data

Trials conducted in the ICU are at high risk of missing data alone on account of the patient's condition [22]. If data are missing, we will consider using multiple imputations according to the recommendations by Jakobsen and colleagues [21]. These recommendations state that up to 40% of missing data can be imputed, but the method of choice depends on the outcomes, whether the dependent variable has missing data only at baseline, etc. [21]. If multiple imputations are used, we will use a worst-best best-worst analysis which for continues data will be based on 2 SD of the mean and for dichotomous data on best and worst. The following variables will be incorporated in the analysis: baseline value of the dependent variable, stratification variable (GCS), end of post-traumatic amnesia, and days to the first mobilization. For all continuous clinical outcomes, we will analyze survivors, and in a sensitivity analysis, impute the lowest possible value for participants who died or dropped out as well as the best possible value. We will present the results of both analyses.

#### 7.1.1 Trial status and profile

The inclusion period ended in December 2018, with only 38 patients included for two years. The end of the three-month follow-up period will be in March 2019, and the one-year follow up will be in December 2019. The flow of patients will be presented in a CONSORT diagram, as reported in the protocol [1]. We will report the number of screened patients, the number of included patients, and the main reason for the exclusion of eligible patients. Furthermore, we will present the number of patients who died within the four-week intervention period, within the first three months from randomization, and within the first year.

Presentation of results in tables and figures.

For the presentation of tables and figures, please see additional file 2.

## 8 Discussion

This statistical analysis plan for the feasibility trial of conducting early orthostatic exercise in patients with severe traumatic brain injury is published to minimize outcome reporting bias and data-driven results. From the total data gathered in the trial, the primary outcomes are feasibility outcomes, but we have also described assessments of our exploratory outcomes.

Our statistical analysis plan is based on considerations to secure unbiased data handling and analyses without getting inspired by the collected data, i.e., *P*-hacking [19]

The use of Trial Sequential analysis for the exploratory clinical outcomes will help establish sample size estimation for a larger trial. One objective of the present trial would direct which outcome to choose. Assessing the functional outcome in patients with a severe traumatic brain injury throughout illness is challenging since they may present with a reduced level of consciousness in the early stage but may eventually return to work. Hence, the scale must encompass many outcomes. The alternative would be to use a scale such as the Glasgow Outcome Scale Extended. This scale is cruder than other scales, and its validity, while the patient is admitted to a hospital department, may be limited. For future trials, our trial results may inspire the initial sample size calculation, which can then be adjusted as data from more trials are added.

Our statistical analysis plan has some limitations. The analysis plan was finished before we began the data analysis. Due to several unforeseen events, the original analysis plan was not published immediately, which would have been optimal. We did, however, manage to make the original analysis plan publicly available. Furthermore, multiple imputations for missing data assumes that these are missing at random; however, this assumption may be incorrect. For example, data completeness may differ between patients in the intervention and the standard care group.

## 9 Conclusions

The HUT-TBI trial investigates the feasibility of early orthostatic exercise versus usual care. With the present pre-specified statistical analysis plan, we hope to minimize analytic bias. On the larger scale, we hope that the feasibility outcomes and the exploratory outcomes may inform and enable the generation of hypotheses for a larger multicenter trial investigating the benefits and harms of early orthostatic exercise.

## Funding

The trial has been funded by “The Council of Danish Victims Fund” (grant 16–910-00043), by the “Research Fund of Rigshospitalet, Copenhagen University Hospital” (R114-A4672), and the “Danish Physical Therapy Association” (15242). The funders did not influence the design of the trial.

## Availability of data and materials

The datasets analyzed during the current study are not publicly available due to the small sample size but are available from the corresponding author on request.

## Authors’ contributions

All authors were involved in the conception of the statistical analysis plan. CGR drafted the statistical analysis plan. CGR, JCJ, CO, JM, and KM provided input for drafting and finalizing the statistical analysis plan. JCJ acted as a senior statistician and CO as a co-statistician. JCJ and CO did the analysis independently. KM is the chief investigator of the trial. All authors read and approved the manuscript for publication.

## Consent for publication

Not applicable.

## Data management plan and standard operating procedure

The data management plan and standard operating procedure are kept at the Copenhagen Trial Unit.

## Declaration of competing interest

The authors declare that they have no known competing interests.
