# Supplementary material for: Statistical analysis plan: Early mobilization by head-up tilt with stepping versus standard care after severe traumatic brain injury
Source: Contemp Clin Trials Commun. 2021 Nov 15;24:100856. doi: 10.1016/j.conctc.2021.100856 (PMC8628210; doi:10.1016/j.conctc.2021.100856)
Supplement: Multimedia component 1 [file mmc1.docx]

|  | **Standard deviation** | **Minimal relevant difference** | **Alpha** | **Assumed sample size** | **Power** |
| --- | --- | --- | --- | --- | --- |
| **CRS-R** | 6 | 3* | 5.0% | 38 | 31.5% |
| **EFA** | 6 | 3** | 5.0% | 38 | 31.5% |
| **FIM** | 20 | 9*** | 5.0% | 38 | 31.5% |
| CRS-R: Coma Recovery Scale-Revised; EFA: Early Functional Ability scale; FIM: Functional independence measure.  * The standard deviation of the CRS-R was estimated from the change value of two studies and used to estimate the minimal relevant difference (SD/2) [[21](#_ENREF_21), [22](#_ENREF_22)]  ** The standard deviation of the EFA score was estimated from two observational studies investigating patients with brain injury approximately 1.5 months after injury and used to estimate the minimal relevant difference (SD/2) [[23](#_ENREF_23), [24](#_ENREF_24)]  *** The FIM standard deviation and the minimal relevant difference has been investigated in one study on patients with brain injury [[12](#_ENREF_12)] | | | | | |
