# Supplementary material for: Statistical analysis plan: Early mobilization by head-up tilt with stepping versus standard care after severe traumatic brain injury
Source: Contemp Clin Trials Commun. 2021 Nov 15;24:100856. doi: 10.1016/j.conctc.2021.100856 (PMC8628210; doi:10.1016/j.conctc.2021.100856)
Supplement: Multimedia component 2 [file mmc2.docx]

**Figures and tables for publication I**

**Table 1. Baseline characteristics of included participants**

|  | Early orthostatic exercise (n=) | Usual care group (n=) |
| --- | --- | --- |
| Age (years) – median (IQR) |  |  |
| Male – n (%) |  |  |
| Brain injury (initial CT-scan) – n (%) |  |  |
| tSAH  aSDH |  |  |
| cSDH  EDH |  |  |
| IVH |  |  |
| Contusion |  |  |
| Mechanism of injury – n (%) |  |  |
| Traffic |  |  |
| Fall |  |  |
| Blunt force |  |  |
| Suicide attempt |  |  |
| Unknown |  |  |
| Secondary injury – n (%) |  |  |
| 1 fracture of extremities or trunk |  |  |
| > 1 fracture of extremities or trunk  No fractures |  |  |
| Comorbidities – n (%) |  |  |
| Diabetes (type II)  Pulmonary heart disease |  |  |
| Hypertension |  |  |
| Schizophrenia  Chronic obstructive lung disease  Atrial fibrillation |  |  |
| None |  |  |
| Neurosurgical procedures performed – n (%) |  |  |
| Evacuation of hematoma |  |  |
| Craniotomy |  |  |
| Craniectomy |  |  |
| External ventricular drain |  |  |
| VP shunt |  |  |
| First measured GCS – median (IQR) |  |  |
| GCS at inclusion - n (%) |  |  |
| Low GCS (3 to 6) |  |  |
| High GCS (7 to 10) |  |  |
| Sedated at randomization – n (%) |  |  |
| RASS– median (IQR) |  |  |
| Days from injury to randomization – median (IQR) |  |  |
| Days to first mobilisation - median (IQR) |  |  |
| Days at the Neurointensive Care Unit – median (IQR) |  |  |
| Days at the RU – median (IQR) |  |  |
| End of PTA (days) – median (IQR) |  |  |
|  |  |  |

Legend: SD: Standard deviation; n: number; tSAH: traumatic subarachnoid haematoma; aSDH: acute subdural haematoma; cSDH: chronic subdural haematoma; EDH: epidural haematoma; IVH: intraventricular haematoma; TBI: traumatic brain injury; EVD: extra ventricular drain; VP shunt: ventriculoperitoneal shunt; GCS: Glasgow coma score; IQR: Interquartile range; RASS: Richmond agitation sedation scale; NCCU: Neurocritical care unit; RU: Rehabilitation unit; PTA: Posttraumatic amnesia;

**Table 2. Feasibility outcome**

|  | n/N (% [95% CI]) *(Wilson intervals)* | |  |
| --- | --- | --- | --- |
| Included patients |  | |  |
| Patients with >60% completed exercises |  | |  |
|  | Early orthostatic exercise (n=) | Usual care group (n=) |  |
| Orthostatic exercises performed – mean (±SD) |  |  |  |
| Additional mobilizations – median (IQR) |  |  |  |
| Additional mobilisations by nurses - median (IQR) |  |  |  |

Legend: N: All patients; 95%CI: 95% confidence interval; SD: standard deviation; IQR: interquartile range;

**Table 3. Adverse events and reactions after intervention period (4 weeks)**

|  | Early orthostatic exercise (n=) | Usual care group  (n=) |
| --- | --- | --- |
| **Number of events** |  |  |
| Adverse events – n (%) |  |  |
| Serious adverse events – n |  |  |
| Adverse reactions – n |  |  |
| Serious adverse reactions - n |  |  |
| SUSAR - n |  |  |
| **Patients experiencing at least one** |  |  |
| Adverse events – n (%) |  |  |
| Serious adverse events – n |  |  |
| Adverse reactions – n |  |  |
| Serious adverse reactions - n |  |  |
| SUSAR - n |  |  |

Legend: n: number; SUSAR: Suspected unexpected serious adverse reaction;

**Table 4. Adverse events and reactions after intervention period (4 weeks) – Per protocol analysis**

|  | Early orthostatic exercise (n=) | Usual care group  (n=) |
| --- | --- | --- |
| **Number of events** |  |  |
| Adverse events – n (%) |  |  |
| Serious adverse events – n |  |  |
| Adverse reactions – n |  |  |
| Serious adverse reactions - n |  |  |
| SUSAR - n |  |  |
| **Patients experiencing at least one** |  |  |
| Adverse events – n (%) |  |  |
| Serious adverse events – n |  |  |
| Adverse reactions – n |  |  |
| Serious adverse reactions - n |  |  |
| SUSAR - n |  |  |

Legend: n: number; AE: Adverse event; SAE: Serious adverse event; AR: adverse reaction; SAR: Serious adverse reaction; SUSAR: Suspected unexpected serious adverse reaction;

**Figure 2. Exploratory outcomes.**

**
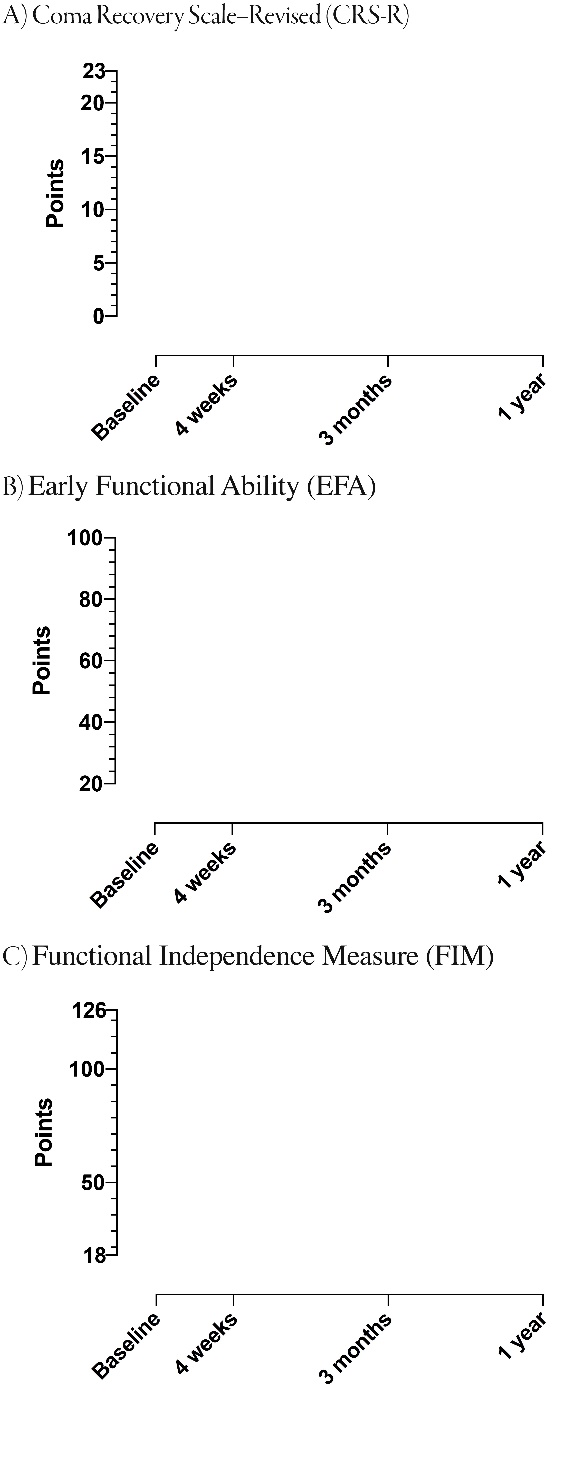
**

Legend: Figure displays score obtained within the two treatment groups at baseline, after 4 weeks, 3 months and, one-year.

**Supplementary table 1. Exploratory clinical outcome for baseline, end of intervention, three months and one year – Intention-to-treat.**

|  | Usual care group | | |  | | Early orthostatic exercise | | |  | |
| --- | --- | --- | --- | --- | --- | --- | --- | --- | --- | --- |
|  | Baseline | End of intervention | Three months from injury | | One-year follow-up | Baseline | End of intervention | Three months from injury | | One-year follow-up |
| **CRS-R** (N) |  |  |  | |  |  |  |  | |  |
| Median (IQR) |  |  |  | |  |  |  |  | |  |
| **EFA** (N) |  |  |  | |  |  |  |  | |  |
| Median (IQR) |  |  |  | |  |  |  |  | |  |
| **FIM** (N) |  |  |  | |  |  |  |  | |  |
| Median (IQR) |  |  |  | |  |  |  |  | |  |
| **GOSE** (N) |  |  |  | |  |  |  |  | |  |
| Median (IQR) |  |  |  | |  |  |  |  | |  |

**Supplementary table 2. Exploratory clinical outcome for baseline, end of intervention, three months and one year – Per protocol.**

|  | Usual care group | | |  | | Early orthostatic exercise | | |  | |
| --- | --- | --- | --- | --- | --- | --- | --- | --- | --- | --- |
|  | Baseline | End of intervention | Three months from injury | | One-year follow-up | Baseline | End of intervention | Three months from injury | | One-year follow-up |
| **CRS-R** (N) |  |  |  | |  |  |  |  | |  |
| Median (IQR) |  |  |  | |  |  |  |  | |  |
| **EFA** (N) |  |  |  | |  |  |  |  | |  |
| Median (IQR) |  |  |  | |  |  |  |  | |  |
| **FIM** (N) |  |  |  | |  |  |  |  | |  |
| Median (IQR) |  |  |  | |  |  |  |  | |  |
| **GOSE** (N) |  |  |  | |  |  |  |  | |  |
| Median (IQR) |  |  |  | |  |  |  |  | |  |
